# Emotional state as a modulator of autonomic and somatic nervous system activity in postural control: a review

**DOI:** 10.3389/fneur.2023.1188799

**Published:** 2023-08-31

**Authors:** Karlee J. Hall, Karen Van Ooteghem, William E. McIlroy

**Affiliations:** Department of Kinesiology and Health Sciences, University of Waterloo, Waterloo, ON, Canada

**Keywords:** emotional state, emotional response, emotion, feeling, autonomic nervous system, somatic nervous system, limbic system, postural control

## Abstract

Advances in our understanding of postural control have highlighted the need to examine the influence of higher brain centers in the modulation of this complex function. There is strong evidence of a link between emotional state, autonomic nervous system (ANS) activity and somatic nervous system (somatic NS) activity in postural control. For example, relationships have been demonstrated between postural threat, anxiety, fear of falling, balance confidence, and physiological arousal. Behaviorally, increased arousal has been associated with changes in velocity and amplitude of postural sway during quiet standing. The potential links between ANS and somatic NS, observed in control of posture, are associated with shared neuroanatomical connections within the central nervous system (CNS). The influence of emotional state on postural control likely reflects the important influence the limbic system has on these ANS/somatic NS control networks. This narrative review will highlight several examples of behaviors which routinely require coordination between the ANS and somatic NS, highlighting the importance of the neurofunctional link between these systems. Furthermore, we will extend beyond the more historical focus on threat models and examine how disordered/altered emotional state and ANS processing may influence postural control and assessment. Finally, this paper will discuss studies that have been important in uncovering the modulatory effect of emotional state on postural control including links that may inform our understanding of disordered control, such as that observed in individuals living with Parkinson’s disease and discuss methodological tools that have the potential to advance understanding of this complex relationship.

## 1. Introduction

Postural control requires the centre of mass (COM) of an individual to be maintained within the limits of the base of support (BOS). It is a complex function involving peripheral, spinal and supraspinal signaling and processing as well as finely tuned and precisely executed motor output. This maintenance of posture requires homeostasis of many different systems of the body and the integration of multi-level nervous system inputs and processing. Research has shown that the motor system is influenced by sensory, cognitive, and behavioral state inputs (1) and thus the state of an individual’s nervous system at the time of the executed movement or postural response influences the motor output of the system. Emotional states (e.g., anxiety, fear) can be considered the product of an emotional response and the associated conscious feeling. Individuals’ emotional state influences their perception of sensory stimuli, with more threatening perceptions of sensory inputs evoked during fearful states (2, 3) (see Table 1 for definitions of relevant terms). Historically, the most common emotional states probed in the postural control literature have been fear and anxiety (4–8). There is strong evidence of links between autonomic nervous system (ANS) activity and somatic nervous system (somatic NS) activity during postural control activities (4, 5, 9–12). The ANS is made up of the parasympathetic and sympathetic divisions (PNS and SNS) and is responsible for automatic bodily processes such as breathing, heart rate and digestion. Research has demonstrated that the ANS and somatic NS share key “relay stations” in the brainstem, cerebral cortex, and basal ganglia (13–15) and these regions are connected to and influenced by a distributed network of brain regions responsible for emotional processing, the limbic system. There are many different examples of tasks which routinely require the coordinated efforts of the ANS and somatic NS that help to shed light on the importance of the neurofunctional link between these systems. Postural stability requires a dynamic interplay between emotional state, self-awareness, attention allocation, autonomic activity and sensorimotor processing, even under conditions which may be considered automatic (16). As such, it is imperative that balance assessment in both research and clinical settings consider an individual’s emotional state.

**Table 1:** Definitions of relevant terms.

Autonomic Nervous System (ANS)	Consists of parasympathetic and sympathetic divisions (PNS and SNS). Responsible for bodily processes such as breathing, heart rate and digestion. Regulates “fight-or-flight” and “rest and digest” responses.
Somatic Nervous System (somatic NS)	A component of the peripheral nervous system. Plays a role in voluntary movements and sensory processing.
Emotion/Emotional Response	The set of physiological responses that occur when the central nervous system (CNS) detects certain negatively or positively valanced stimuli. Responses are coordinated through neural substrates that comprise the limbic system and ANS. Responses include changes in arousal level in the CNS, cognitive function, and autonomic, endocrine, and musculoskeletal responses.
Feeling	The conscious experience of emotions/emotional responses.
Cognition	The mental action or process of acquiring knowledge and understanding through thought, experience, and the senses (includes processes such as attention, perception, sensation).
Emotional State	Emotion/emotional response and the associated conscious feeling.
Cognitive-Emotional State	Encompasses both the emotional state and the associated cognitive contributions to the individual’s state of being (i.e., sensation, perception, attention).
Central Set	The state of readiness of the nervous system for an impending act, based on prior experiences and perception of external conditions.
	Proposed definition: The state of a readiness for an impending act based on the *cognitive-emotional* state of an individual’s nervous system at any given instant in time.
	Affect	The external expression of emotion, often expressed in facial or bodily reactions and speech.
	Mood	A persistent emotional state, or in other words, the internalized state of emotions and their associated feelings.
	Threatening Perception	Sensory inputs that an individual consciously or subconsciously perceives as threatening.
Anxiety	Emotional state caused by external or internal stimuli and that underlies a specific set of measurable behavioural, physiological, hormonal, and autonomic reactions, as well as a consciously perceived feeling/experience (subjective feeling of anxiety/ worry, nervousness, or unease). Can be transient or persistent.
Fear	Emotional state caused by external or internal stimuli that an individual has perceived as threatening or dangerous (subjective feeling of fear) and that underlies a specific set of measurable behavioural, physiological, hormonal, and autonomic reactions. Can be transient or persistent.

To explore how emotional states affect standing balance control, researchers have taken various approaches, from experimentally manipulating fear or anxiety using a threat model to studying postural sway in individuals with varying levels of anxiety or fear. Studies have also revealed the importance of emotional state on dynamic tasks such as gait initiation (17, 18). Due to the challenge in distinguishing the influence of emotional state on postural control versus voluntary movement initiation in gait initiation, the current review focusses primarily on studies that evaluate control of postural equilibrium in spontaneous and/or perturbed standing balance tasks. Furthermore, in an effort to maintain this focus, we do not discuss anticipatory postural adjustments in this paper. This area of study is broadly relevant for understanding postural control in order to effectively identify, address and prevent instability that may contribute to increased risk of falls and/or reduced mobility. The objectives of this paper are to:

1. Outline a conceptual framework of common terms as they pertain to the effects of emotional states on postural control.
2. Briefly review neuroanatomical links between emotion processing, and ANS and somatic NS control of posture.
3. Review physiological and behavioural evidence of the association between emotional state, ANS activity, somatic NS activity and posture control.
4. Explore how disordered/altered emotional or ANS processing may influence posture control and assessment.

## 2. Conceptual framework for studying the influence of emotional state on postural control

The term emotion is commonly used in two distinct ways, either being referred to as a physiological response to stimuli or as the conscious experiences accompanying these bodily responses (often thought of as “feelings”). Furthermore, it is often left undefined in many studies, adding to the confusion. One important aim of this paper is to propose a framework for discussing emotion with regards to postural control (see Table 1 for summary of definitions). Here, the term *emotion* and *emotional responses* is used to refer to the set of physiological responses that occur when the central nervous system (CNS) detects certain negatively or positively valanced stimuli. These are automatic physiological responses that occur both within the cortex and subcortical regions and are expressed in different systems within the body. In cortical and subcortical areas, these emotions involve changes in arousal levels and in cognitive functions such as attention, memory processing and behavioural strategies (19). Within the body, these emotions involve endocrine, autonomic, and musculoskeletal responses (19). LeDoux (20) stated that feelings are “accounts our brain creates to represent the physiological phenomena generated by our emotions.” The term “*feeling*” will be used when referring to the conscious experience of these somatic and cognitive responses. The term “*mood*” can be defined as a persistent emotional state, or in other words, the internalized state of emotions and their associated feelings. The term “*affect*” is the external expression of emotion, often observed in facial or bodily reactions and speech. Conceptually, and for example, fear and anxiety have been regarded as emotional states that are caused by external or internal stimuli and that underlie a specific set of measurable behavioural, physiological, hormonal, and autonomic reactions (21). Fear and anxiety can be either transient or persistent emotional states depending on chronicity, and the outward expression of these emotional states are the individual’s affect. “*Cognition*” is defined as the mental action or process of acquiring knowledge and understanding through thought, experience, and the senses. Cognition includes such mental processes as sensation, attention and perception, and complex operations such as memory, learning, language use, problem solving, decision making, reasoning and intelligence. The concept “*cognitive-emotional state*” encompasses both the emotion and the associated conscious feeling, as well as the associated cognitive contributions to individuals’ state of being. In a review of the relationship between emotion and cognition, Pessoa (22) states that “behaviour is a product of the orchestration of many brain areas; the aggregate function of these brain areas leads to emotion and cognition.” Brain regions viewed as “affective”/emotional are also involved in cognition (and regions viewed as “cognitive” are also involved in emotion), and it is known that cognition and emotion are integrated in the brain. Thus, it is important to reflect this relationship in the conceptual framework we use in this field of study. “*Central set*,” a term used often within postural control research is defined as the state of readiness of the nervous system for an impending act, based on prior experiences and perception of external conditions. However, it is proposed here that central set be redefined as the state of readiness for an impending act, based on the *cognitive-emotional state* of an individual’s nervous system at any given instant in time. This would expand on the already established cognitive contributions to nervous system state to include emotion/emotional response and the associated conscious feeling/experience, as well as the associated cognitive contributions to individuals’ state of being (including sensation, perception, attention, memory of past experience, etc.). It also reflects the physiological responses that occur within cortical and subcortical areas at any instant in time (19). For example, when a loss of balance occurs, an individual’s nervous system is processing/evaluating: (1) somatic motor responses (i.e., processing sensory information during a loss of balance triggers subsequent motor and autonomic reactions aimed at recovering balance), (2) emotional responses (internal and external sensory stimuli trigger emotional responses, including ANS responses and feelings prior to, and during, the loss of balance), and (3) cognitive responses (sensory information results in allocation of attention, elicits retrieval or storage of context specific memories, etc.). All of these processes/responses come together to result in a behavioural response; an action driven by the present sensory information and the individual’s internal state (22).

In the present paper, focus is often directed to the construct of “*emotional state*” (emotion and its associated conscious feeling) as a subset of “*cognitive-emotional state*.” This is done, in part, to integrate historical frameworks/conceptualization of emotion/emotional state as an independent process from cognition. Specifically, the modulatory effects of emotional state on ANS and somatic NS activity during postural control are examined. The potential association between cognitive-emotional state and postural control can be considered in both indirect and direct ways. An indirect association is the influence of system responses to changes in cognitive-emotional state that do not expressly influence the somatic NS processing to control balance. As an example of an indirect influence, increased ventilation that can be associated with increased arousal may influence measures of postural sway. Alternatively, direct influences would reflect neuro-modulatory effects and interactions between somatic NS control of sensorimotor processes for postural control, and cortical and subcortical changes in activity linked to change in cognitive-emotional state. To understand the potential for direct influences on postural control, the following section briefly reviews neural control of emotional responses, including the neural substrates involved and neuroanatomical associations to somatic control systems.

## 3. Associations between neuroanatomical networks for emotion processing, ANS and somatic NS control of posture

Researchers have identified neural links between regions of the brain that are involved in emotional states and those that are responsible for balance control (23, 24). The ANS is thought to be one of the most important mediators between the mind and body/viscera (25). Studies show that feelings of fear and anxiety trigger sympathetic nervous system drive and this manifests as physical symptoms/characteristics (26). Homeostasis is mediated via the ANS by fine tuning the balance between sympathetic, parasympathetic and various hormonal systems (27) and is influenced by a number of cortical regions as well as regions in the brain stem, and peripheral and visceral system inputs. We will briefly review key shared “relay stations” in neuroanatomical networks for emotion processing, ANS and somatic NS below.

**Figure 1:**
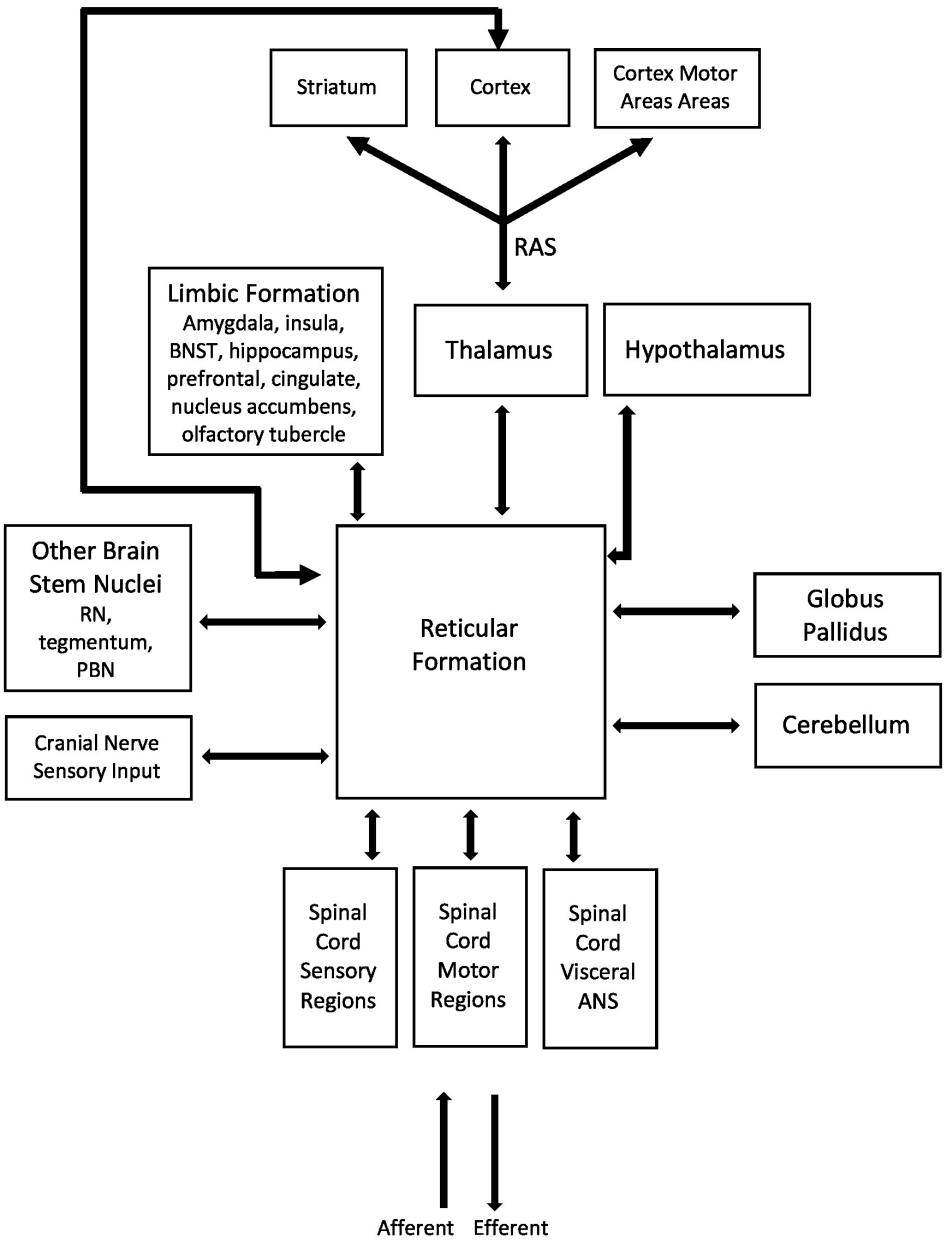
Simplified hypothetical model of the shared neural substrates between the limbic system, autonomic and somatic nervous systems. These shared neural networks and pathways drive the experience of emotional states, autonomic activity and postural control. The reticular formation (RF) has complex connections to multiple regions in the CNS and is a site of convergence, divergence and overlap (filtering input and regulating output), with projections to most nuclei within the brainstem, cortical and subcortical regions including limbic regions and the spinal cord. It is well positioned to be one of the main shared neural substrates for interaction and modulation of emotional state, autonomic and somatic nervous system activity. The RF and reticulospinal tracts main functions are (1) autonomic control, (2) respiration and (3) tone of postural muscles. The limbic system is involved in emotional processing, motivation, learning and memory and operates by influencing the endocrine and autonomic systems. Neural substrates that make up the limbic system have bidirectional connections to the RF and it is likely through these connections that incoming sensory/visceral information can modulate our emotional states and perceived feelings, and how feelings and emotional states can influence altered output through somatic and autonomic nervous systems.

The reticular formation (RF) is a network of nuclei in the brainstem that plays a key integrative role in the relationship between emotional state and ANS and somatic NS activity (Figure 1). The RF has complex connections to multiple regions in the CNS and is a site of convergence, divergence and overlap (filtering input and regulating output), with projections to most nuclei within the brainstem, cortical and subcortical regions including limbic regions and the spinal cord. The three main functions of the RF are (1) autonomic control; (2) respiration and; (3) postural muscle tone. When a stimulus is detected by RF nuclei, system wide lowering of membrane threshold potentials occurs which results in an increase in general nervous system excitability. This has an impact on factors such as reaction time and is likely behind the short latencies we see with balance reactions (28). The reticular activating system (RAS) consists of ascending fibres from the RF which contribute to vital activities in relation to the vigilance state of animals, including humans (29). The RAS receives collaterals from specific ascending pathways. Most ascending fibres of RAS relay in midline and intralaminar nuclei in the thalamus and spread diffusely into various parts of the cerebral cortex, modulating cortical activities via thalamocortical networks (30). The RAS therefore diffusely stimulates the cerebral cortex and along with the thalamus, is responsible for our level of arousal and attention, and modifies postural muscle tone (31). Furthermore, brainstem projections and those descending to the spinal cord contribute to innate motor functions such as eye-head coordination (32, 33) and control of posture and locomotion (34–41) among other functions. It should be noted that much of this research comes from animal models, although there are case studies in individuals with lesions to sections of their RF. Takakusaki et al. (29) report that in most vertebrates, reticulospinal neurons contribute to various types of locomotor movements such as swimming in fishes, crawling in reptiles, flying in birds, quadrupedal locomotion in higher mammals and bipedal gait in higher primates (37). Reticulospinal neurons activate neuronal circuits in the spinal cord that generate locomotor rhythm (central pattern generator) (37, 38, 40–42). Studies have shown the presence of functional topographical organizations with respect to the regulation of postural muscle tone and locomotion in both the mesopontine tegmentum and the pontomedullary reticulospinal system (29). These organizations are modified by neurotransmitter systems, particularly the cholinergic pedunculopontine tegmental nucleus projections to the pontine reticular formation. The author states that because efferents from the forebrain structures as well as the cerebellum converge to the mesencephalic and pontomedullary reticular formation, changes in these organizations may be involved in the appropriate regulation of posture-gait synergy depending on the behavioral context. Furthermore, it is through bidirectional communication between structures of the limbic system and the RF that feelings and emotional states modulate activity of the emotional response, including changes in autonomic activity, breathing rate and postural muscle tone, among other things.

The limbic system is involved in emotion, motivation, learning and memory and operates by influencing the endocrine system and ANS (26, 43). The limbic system consists of six main neural substrates in the brain: (1) the thalamus, which acts as a primary sensory relay station (26, 44); (2) the hypothalamus, which plays a key role in the regulation of many important functions of the body including circadian rhythm and regulation of the ANS; (3) the hippocampus, which plays a key role in forming new memories; (4) the amygdala which rates the emotional importance of the situation, plays a key role in processing emotions and is largely tied to anxiety and fear; (5) the cingulate cortex which plays a role in affect regulation to assist in learning from experience (45, 46); and (6) the basal ganglia which plays a role in habitual behaviour, emotion and cognition (47, 48). In addition, the prefrontal cortex is heavily connected to structures of the limbic system and has been shown to influence both emotive control and movement (49–51). Neural substrates that make up the limbic formation have bidirectional connections to the RF and sharing of information through these connections is one mechanism by which incoming sensory/visceral information can modulate our feelings and emotional states and how our feelings and emotional states can influence altered output through our somatic NS and ANS (muscle tone, heart rate, breathing, etc.). Of important note when discussing the fight or flight response is that the hypothalamus, which integrates a range of sensory inputs and coordinates autonomic, endocrine and behavioural responses aimed at maintaining body setpoints (homeostasis) or overcoming stressors (allostasis), has bidirectional connections to the RF and the thalamus, as well as connections to various other neural substrates of the limbic system (52). As mentioned in Section 2, there are physiological responses that occur when the CNS detects negatively or positively valanced stimuli, and these are mediated in large part by the RF/RAS and hypothalamus. In cortical and subcortical areas, these responses involve changes in arousal levels and in cognitive functions such as attention, memory processing and behavioural strategies. Within the body, these responses involve endocrine, autonomic, and musculoskeletal responses (19). Furthermore, autonomic activity, such as increased respiration or heart rate, has a direct modulatory effect on the activity of the thalamic nuclei (i.e., changing their firing rate) and this may have implications for the way sensory and motor information is processed and/or relayed by the thalamus to other regions of the cortex and subcortical areas during emotional responses (53). This has direct implications for postural control because if the gain of somatosensory processing has been altered, it will result in a change in response to a given stimulus, such as loss of balance.

In summary, research has demonstrated that the ANS and somatic NS share key “relay stations” in the brainstem, cerebral cortex and basal ganglia (13–15) and these regions are part of, or connected to, the limbic system. The coordination/integration of these systems seems to be a key component in postural control and learning through experience, involving the coordination of higher centres, the ANS and the somatic NS. These shared neuroanatomical networks between the limbic system and somatic NS point to the important associations between ANS activity, emotional state and somatic control, and inform the selection of our methodical strategies to concurrently measure emotional state and ANS activity during postural control studies.

## 4. Physiological and behavioural links between emotional state, ANS and somatic NS activity, and postural control

Evidence of the association between emotion, ANS and postural control comes primarily from studies that have measured postural control in response to a change in emotional state. The most common approach is to use a postural threat model, in which individuals are placed at height. In 2018, Adkin and Carpenter provided a review of the evidence which supports the efficacy of using height-induced threat to study the effects of arousal, anxiety, and fear of falling on postural control (54). Manipulating the height of the support surface has been shown to increase perceived consequences of instability and results in changes in postural control proposed to be evidence of threat-related changes in emotional state on postural control (54). Beyond the use of height-induced threat models, researchers have used models such as threat of perturbation (i.e., threat of translating balance platform) and social evaluation threat (i.e., presence of an observer/examiner). These previous studies have been invaluable in paving the way for our understanding of the neural mechanisms of threat-related changes in balance control in healthy individuals. This review will consider these studies along with other evidence of the possible linkages between emotional state, ANS and somatic NS activity and postural control. This includes a focus on different emotional states and the impact of pathology on emotion processing and/or the ANS, and how these states influence postural control. To do so, this section will focus on evidence from various associations between emotion, ANS activity and postural control including:

1. Evidence of integrated ANS and somatic NS activity.
2. Direct and indirect associations between emotional state, ANS activity and postural control.
3. Disordered emotional state, ANS function and links to postural control.

### 4.1. Integrated ANS and somatic NS activity

In the daily life of an individual, ANS activity is routinely coordinated and integrated with somatic motor activity and/or neuroendocrine regulation. This happens in response to some combination of external environmental cues, internal physiological conditions, or centrally generated emotional and cognitive states (55–57). This connectivity between systems is apparent when looking at a select few of the many physiological changes that occur in the body during the “fight or flight” sympathetic response. During this response, changes in firing rate of muscles involved in breathing are seen as respiration rate increases (58) and increases in the cell membrane depolarization rate of the sinoatrial node leads to heart rate increases (59). In addition, there is an increase in both smooth and skeletal muscle tone that occurs in times of increased arousal (60, 61). This mechanism is also responsible for the general increase in muscle tension during emotional states such as anxiety, fear and stress with the intensity and duration of sympathetic system responses varying in response to the triggering stimuli. The intensity and duration of the fight or flight response involves a complex interplay between our emotional state, our experience with a given situation, and the state of our sensory and autonomic nervous systems at the time of the event.

### 4.2. Indirect and direct associations between emotional state, ANS and somatic NS activity and postural control

As mentioned above, the potential association between cognitive-emotional state and postural control can be considered in both indirect and direct ways. An indirect association is the influence of system responses to changes in cognitive-emotional state that do not expressly influence the somatic NS processing to control balance. Alternatively, direct influences would reflect neuro-modulatory effects and interactions between somatic NS control of sensorimotor processes for postural control, and cortical and subcortical changes in activity linked to change in cognitive-emotional state. We can draw from the background knowledge of neural control of emotional responses, including the neural substrates involved and neuroanatomical associations to somatic control systems to aid in our understanding of the potential for direct influences on postural control.

By example, postural control can be indirectly altered by emotional state influences on cardiovascular and respiratory outputs that would impact activation of core musculature, as well as movement of the centre of mass as the rib cage expands and retracts with heavy breathing. Furthermore, various emotional states can result in changes to the attentional resources available for allocation to postural control. This can indirectly result in altered balance and posture responses, which have been reflected in changes in amplitude and frequency of center of pressure displacement, as well as measures such as sway area and sway variability (62–65). In contrast, there are proposed direct links between ANS and somatic NS activity during the control of balance, specifically during reactive balance control most commonly by exploring the impact on postural control associated with changes in fear, anxiety through changes in postural threat (4–8). Fear influences the perception of several sensory stimuli, with more threatening perceptions evoked during fearful states (2, 3). In the context of postural control, anxiety has been shown to have a modulatory effect on both ANS and somatic NS activity during quiet stance and during balance reactions (4–6, 9–12, 66–69). It is well established that emotional responses involve changes in sympathetic and parasympathetic activity (58) and there is also strong evidence for ANS activity modulation in balance reaction and postural control (4, 9–12). Researchers have approached this topic by looking into various emotional states and the response elicited by the motor system. Specifically, studies have examined postural threat scenarios where individuals are perturbed while on an elevated platform, and quiet standing and balance tasks in individuals with high versus low trait anxiety. These studies are reviewed below.

Furthermore, evidence suggests the context of the postural threat greatly affects the demonstrated behaviours that are observed. Several studies have examined the impact of context on postural responses to altered emotional states, including the influence of anxiety or threat on core muscle activation, spinal reflex excitability, conscious perceptions of sway as well as others looking at the influence of threat on allocation of attention. These studies have been important in furthering our understanding of the relationship between emotional state, ANS activity and postural control, through the lens of the impact of arousal on postural control outcome measures. However, it is important to build on these findings and embrace a “*cognitive-emotional state*” framework when interpreting the results of these studies, and to help synthesize future methodological approaches to probe these research questions.

If we are to use this research to inform both laboratory studies, and clinical assessment and treatment approaches in the future, it is important to take both an open focus (looking at the overall cognitive-emotional state of an individual and how altered states of being may impact observable behavioural outcomes) as well as a “spotlight” focus on these various specific direct and indirect mechanisms (i.e., looking at the association of cognitive-emotional state on specific processes, such as altered attentional focus and postural control, for example) by which cognitive-emotional state can modulate postural control. As discussed earlier in the paper, brain regions viewed as emotional are also involved in cognition (and regions viewed as cognitive are also involved in emotion), and it is known that cognition and emotion are integrated in the brain (22). The cognitive contributions to an individuals’ state of being (including sensation, perception, attention, memory of past experience, etc.) impact the response of that individuals’ nervous system to a given stimulus (such as a loss of balance/challenge to balance) and stimulus driven changes to cognition (which are integrated with emotional responses), including both altered sensory processing and attentional focus for example (not one exclusively as is often interpreted), can help to clarify many of the findings in this body of literature. When a threat (or an altered emotional state such as anxiety) is present, RF/RAS, thalamus and hypothalamus mediated changes in emotional responses (including endocrine and ANS activity changes) and cognitive processes occur which ultimately manifest as changes in behavioural outcome measures, including altered COP sway and frequency, for example. That is, the interaction between emotional and cognitive responses (including perception of threat) ultimately leads to the context specific behavioural outcome. The next several sub-sections will discuss some of these direct and indirect associations between cognitive-emotional state, ANS activity and postural control in various behavioural studies.

#### 4.2.1. Indirect and direct associations between emotional state and breathing rate and core muscle activation and in quiet stance

##### 4.2.1.1. Association between anxiety, breathing rate and core muscle activation

Studies linking emotional states such as anxiety and fear with autonomic, respiratory and cardiac activity have helped to clarify the neurophysiological relationship between feelings, emotional states and their physical manifestation. It has been demonstrated that stress and anxiety have a modulatory effect on ANS activity and on breathing rate (70–72). Furthermore, irregular breathing involving shallow and/or deep rapid breathing patterns and periods of apnea, has been shown to modulate the ANS leading to excitation of the nervous system (70, 73, 74). With regards to motor control, research has shown that breathing pattern can alter the effectiveness of core musculature used for functional tasks (75–77). This is likely due to the diaphragm, a key breathing muscle, also acting as a key core muscle regulator (78–80) with an associated postural function (81). The diaphragm has been found to contract prior to initiation of upper extremity movement (82, 83) independently of respiration phase (83). When respiration becomes quick and shallow, the diaphragmatic muscle activity is altered, and this is likely to have an impact on how effectively it, and the other core muscles, stabilize the trunk during functional movement (84). In these instances, postural muscles are affected by emotional states indirectly, whereby emotional state and increased sympathetic activity impacts breathing rate, which in turn affects core muscle function. The relationship between pattern of diaphragmatic breathing and core stability has been linked to static and dynamic balance and it has been shown that the amplitude of COP displacement increases with increasing respiration (25, 27, 84–87). While it is possible to argue for a stabilizing benefit of increased trunk stiffness, the more transient impact of changes in ventilation can result in an increase in measured postural sway due to trunk motion (88, 89).

##### 4.2.1.2. Associations between anxiety, stress, and postural sway in quiet stance

Evidence of the modulating influence of arousal during quiet stance was first demonstrated in a 1996 study by Maki et al. (90). Since then, studies looking at postural sway have found that high anxiety modifies the location and frequency of centre of pressure (COP) in the antero-posterior (AP) axis (5, 6). A study by Wada et al. (6) demonstrated increased postural sway in the AP axis in a high versus low trait anxious group. In another study, Bolmont et al. (5), demonstrated that a deterioration in mood state, measured by the profile of mood state (POMS) questionnaire, affects balance performance and participants’ ability to use input from either the somatosensory, visual or vestibular system to maintain balance during quiet stance (5). A study by Hainaut et al. (66), found that when vision was absent, there was an increase in postural sway during static balance control when comparing individuals with high versus low trait anxiety. These authors also suggest that state anxiety could modify the processing of various sensory inputs involved in balance control regardless of the individual’s trait anxiety. Furthermore, a study by Coco et al. (67), revealed a significant positive correlation between cortisol awakening response (marker of chronic stress) and perceived stress, and that these factors influenced postural stability, which manifested as increased COP excursion (67–69). This study also demonstrated a stronger influence of stress when no visual information was present (67). These studies highlight the relationship between emotional state and postural control in quiet stance that may be amplified in more challenging conditions such as reduced vision. One explanation for the altered sensory utilization could be due to the fact that the chronic increased sympathetic activation that is present in those with higher trait relative anxiety, as well as during state anxiety (i.e., situational specific “stress-response”/increased arousal), both mediated by the RF/RAS, thalamus and hypothalamus, leads to changes in sensory processing and subsequent changes in sensory-motor responses (53). As mentioned earlier in this review, this has direct implications for postural control because if the gain of somatosensory processing has been altered, it will result in a change in response to a given stimulus (i.e., loss of balance or challenge to the balance system).

#### 4.2.2. Indirect and direct associations between postural threat models, emotional state and postural control

##### 4.2.2.1. Postural threat studies to increase fear, anxiety and arousal

To gain insight into the relationship between anxiety, ANS and somatic NS activity and postural control/reactions, researchers have experimentally manipulated fear or anxiety by eliciting arousal scenarios with “threats” to individuals, often accomplished by having the participants stand on elevated platforms. Research investigating the effect of height-induced postural threat has shown increased anxiety and fear of falling, lower balance confidence, elevated physiological arousal (as measured by electrodermal activity—EDA) and changes in blood pressure (9–11, 91–94). Researchers have also shown that state anxiety caused by actual threat of a fall could lead healthy participants to develop a postural stiffening strategy characterised by smaller amplitude and higher frequency of COP displacement (4, 7, 95, 96). These and other studies have shown that this postural threat modifies the speed and amplitude of postural sway in quiet standing (4, 7, 9–12). It could be argued that this type of postural anxiety/threat scenario could reflect the possible arousal that a fearful or anxious individual may experience with a loss of balance in real-world settings, and thus has been frequently used as an experimental model to look at the effect of arousal on human balance and related measures of interest. It remains important to keep in mind that this experimental design has context specific consequences to the behavioural outcome (for example, in the elevated platform model participants lean away from the edge, and this impacts COP trajectories). Thus, the context specific changes to behavioural outcomes always needs to be top of mind when interpreting results. As mentioned, it is important to probe these specific relationships to gain an understanding of the indirect and direct associations with emotional state and postural control, however, it remains important to frame these study findings within the lens of a cognitive-emotional state framework. That is, to keep in mind that the interaction between both emotional and cognitive processes and responses ultimately leads to the context specific measurable behavioural outcomes.

##### 4.2.2.2. Postural threat studies (to increase fear, anxiety and arousal) and spinal reflexes

Some studies using the elevated height model have explored the potential influence of postural threat, when standing at height, on spinal reflex excitability of the plantar flexors. It has been proposed that the gain of these reflexes may have an influence on control of standing balance, with higher gain resulting in tighter control. Sibley et al. (97) explored this using the electrically evoked H-reflex and Horslen et al. (98) evaluated both the H-reflex and the mechanically evoked tendon reflex (T-reflex). More recently Hodgson et al. (99) compared H-reflexes during standing at different visual heights, using virtual reality. The results of these studies were mixed, with respect to the H-reflex gain. Sibley et al. (97) and Hodgson et al. (99) revealed a lower gain and Horslen revealed no change in H-reflex standing at height, but instead revealed a higher T-reflex gain which they proposed was linked to higher fusimotor drive. The challenge comparing such studies is the numerous factors, unrelated to postural threat, that may be associated with changes in reflex gain. For example, the evoked reflexes are themselves destabilizing, serving as a perturbation and the observation of reduced gain, while potentially linked to increased threat, could have reflected the CNS attempt to minimize the destabilizing influence of the evoked reflex. In addition, H reflexes are attenuated in more challenging task conditions, unrelated to threat, that is proposed to be linked to reducing sensory inputs to optimize information processing. The speculation of increased fusimotor drive, that maybe linked to threat and the desire to tighten control and reduce sway, is consistent with studies revealing task related differences in fusimotor drive during challenging locomotor tasks. While such reflex studies do afford the potential to reveal insight into the underlying neurophysiological changes that may be linked to emotional state, they will require far greater attention the factors that impact reflex gain including attention to time-varying changes in state that occur during continuous standing.

##### 4.2.2.3. Associations between emotional state, sensory processing and postural control

Two studies by Cleworth and colleagues have probed the question of whether threat has an impact on sensory perception during balance tasks, further looking into a direct association between emotional state, ANS activity and postural control. In one study by Cleworth et al. (100), examining how changes in threat influenced conscious perceptions of postural sway in the antero-posterior plane, participants reported an increased level of fear, anxiety, and arousal, and a decreased level of balance confidence when standing at height (100). These researchers also found that sway amplitude is reduced, while sway perception appears to remain unchanged. They argue that as threat is increased, sensory gain may be increased to compensate for postural strategies that reduce sway (i.e., stiffening strategy), allowing availability of sufficient afferent information to maintain, or even increase the conscious perception of postural sway (100). In another study by Cleworth et al. (101) examining the impact of height-related threat on voluntary balance control (i.e., leaning towards targets) in healthy individuals, it was found that an elevated platform height resulted in significantly increased EDA, fear and anxiety, and decreased balance confidence and that the psychological state of an individual can significantly affect perceived body position during postural tasks (101). Although sensory gain is one mechanism by which threat modulates postural strategies, conscious perception of postural sway, and perceived body position during postural tasks, it is important to consider other possible mechanisms for these results, such as altered attentional focus that would occur when an individual is asked to lean towards targets while a threat is present.

##### 4.2.2.4. Associations between emotional state, attentional allocation and postural control

There is considerable evidence demonstrating the attentional resources required for processing emotional stimuli (102, 103), though it is not clear the specific impact of the emotional state or the underlying cognitive processing. Emotional state is well recognized as having a significant influence on cognitive processes and is specifically influential on attention (102). The “emotional attention” that can be required under different states can compete with other demands on attentional processing. This raises the possibility that changes in postural control associated with changes in emotional state could be linked to altered attentional demands, in some ways paralleling dual-task effects. In such a case, a demanding postural task (i.e., significant need for executive resources), would be more likely to be impacted by emotion processing. Researchers have proposed that changes in allocation of attention may influence the relationship between postural threat (eliciting anxiety) and balance control. The presence of perceived threat can alter allocation of attention, either directing attention toward or away from an individual’s posture, depending on the nature of the threat.

Huffman et al. (104) demonstrated that with height-induced threat, individuals have a greater tendency to consciously control and monitor their posture. This increase in conscious control was shown to be related to leaning further away from the platform edge, independent of any changes in amplitude or frequency of COP displacements. In various studies, when standing in conditions of increased threat, individuals self-report broad shifts in attentional focus such as directing more attention to the mechanics of movement, threat-related stimuli, and strategies to improve confidence and/or reduce anxiety (104–107). These shifts in attention have been associated with, and may indirectly contribute to, specific threat-related changes in standing balance (104–107). For example, recent work revealed that changes in ankle muscle co-contraction and high-frequency COP displacements were the only changes in standing balance to adapt following repeated exposure to height-related threat, with changes in attention to movement as the strongest predictor of changes in high-frequency COP displacements (106). These findings align with research suggesting a relationship between conscious movement control and high-frequency COP displacements in patients with anxiety-related balance disorders (62–64). A study by Johnson et al. (108), found that when participants were threatened, they were more anxious and reported directing more attention to movement processes, threat-related stimuli, and self-regulatory strategies, and less to task-irrelevant information. It was also found that postural sway amplitude and frequency increased with threat. Furthermore, greater increases in frequency and smaller increases in amplitude of COP were observed with perturbation experience. In instances with no experience, it was found that threat-related changes in postural control could be accounted for by changes in anxiety, with larger changes in anxiety related to larger changes in sway amplitude. The authors state that with perturbation experience, threat-related postural control changes were accounted for by changes in attentional focus, with increases in attention to movement processes being related to greater forward leaning and increases in sway amplitude. It was also found that increases in attention to self-regulatory strategies were related to greater increases in sway frequency. These findings suggest relationships between threat-related changes in anxiety, attentional focus, and postural control depend on the context associated with the threat.

Although changes in attentional focus is one mechanism by which threat modulates postural strategies, changes in sensory processing/sensory gain of information that is relevant to the balance task plays a role in shifts in attentional focus that may occur. For example, recent research in healthy older adults has shown that conscious balance processing may drive behaviours that are opposite to postural stiffening responses (i.e., reduced sway frequency and increased sway amplitude) (109, 110). In young healthy adults, conscious movement processing (CMP) has been shown to increase sway amplitude during relatively simple static balance tasks (111, 112). However, as task difficulty increases, the effect of CMP on postural sway may change, with some evidence to suggest that CMP may help enhance balance performance (110, 113). This could be due in part to increased sensory gain to task-relevant sensory information and it can be reasoned that sensory gain changes (i.e., increased processing of internal sensory information pertaining to control of movement) the more an individual shifts attentional focus into conscious control of their movement. Jie et al. (114) argues that compared to a low-CMP condition, high CMP (as often seen in anxious individuals and clinical populations) leads to increased postural sway, which is often interpreted as worse performance in an easy, very stable (solid surface) task condition. However, these effects may be less pronounced or even reversed during more challenging balance conditions in which individuals are standing on more unstable surfaces, such as foam (114). The challenge of standing on foam, depending on the compliance, is an alteration in the sensory input that arises from plantar surface contact and but also change in the transfer of muscle force to the support surface. This will impact both the required sensorimotor transformation and the attentional focus which may account for differences compared to stable support surface conditions.

##### 4.2.2.5. Association between repeated exposure to height induced threat, emotional state and postural control

In a study by Zaback et al. (115), individuals were repeatedly exposed to height-induced postural threat to determine if reducing the emotional response to threat influences standing balance control. Following repeated threat exposure, participant emotional responses to the threat were attenuated, however, the threat-induced changes in standing balance were still present. When initially exposed to the threat, individuals leaned backward (a context specific direct association) and demonstrated smaller amplitude and higher frequency of COP adjustments and these balance outcome measures did not change following repeated threat exposure. High frequency COP oscillations (>1.8 Hz) and ankle muscle co-contraction showed adaptation to repeated exposure to the threat, with behavioural adaptations being accounted for by a combination of emotional and cognitive state changes. The findings suggest that some threat-induced standing balance changes are more closely linked with the arousal response to the threat than others and therefore, are altered by the repeated exposure to the threat. The arousal response is one aspect of an emotional response, in addition to the RF/RAS, thalamus and hypothalamus mediated changes in cognitive processing. As cognitive processes and emotional processes in the brain are integrative, it is the interaction between emotional (including arousal) and cognitive processes will result in the observed behavioural outcomes. Other findings reflect the context specific conditions that the task imposes, which ultimately also contribute to the observed behavioural outcome. In a follow up study, Zaback et al. (116) used a prolonged threat exposure protocol to manipulate emotional state within a threatening context to determine if any threat-induced standing behaviours are employed independent of emotional state. With the initial threat exposure, individuals leaned backward, showed reduced low-frequency COP power, and increased high-frequency COP power as well as plantar/dorsifexor coactivation. Following repeated exposure, the psychological and autonomic responses to the threat were decreased and high-frequency COP power and plantar/dorsifexor coactivation habituated. In this study, individuals were re-exposed after 2–4 weeks and demonstrated a partial recovery of the emotional response to the threat with few standing balance adaptations retained. The authors suggested that some threat-induced standing behaviours are coupled with the psychological and autonomic state changes induced by the presence of threat, while others may reflect context-appropriate adaptations resistant to habituation.

In another study examining the effects of initial and repeated postural threat exposure on emotional, cognitive, and postural responses, Johnson et al. (117) had young and older adults stand on a force plate fixed to a translating platform and manipulated threat through expectation of temporally and directionally (left or right) unpredictable platform perturbations. Postural threat elicited similar emotional, cognitive, and postural changes in young and older adults. With initial threat exposure, participants reported increases in self-reported anxiety and physiological arousal, as well as broad changes in attentional focus. Participants also significantly increased COP amplitude and frequency, and COP power within medium and high frequencies. With repeated threat exposure, anxiety, arousal, and some threat-induced changes in attentional focus significantly adapted. The authors reported that changes were accompanied by significant reductions in COP frequency and COP power within medium frequencies. Some emotional and cognitive outcomes returned to no threat levels while postural outcomes did not. The authors concluded, similarly to the conclusions made by Zaback et al. (115), that their findings suggest that some threat-related changes in standing postural control may be closely linked with one’s emotional response to threat, while others may be context-dependent. When participants experienced the feeling of anxiety and physiological arousal, there is an internal shifting of attentional focus, potentially through mechanisms associated with RAS/RF mediated changes in cognitive processes. The associated altered COP frequencies may reflect both the RF/ANS meditated changes in attentional focus, postural tone and altered sensory perception gain with changes in cognitive-emotional state.

##### 4.2.2.6. Associations between threat, conscious experience (feeling) of fear and postural control

Ellmers et al. (118), has proposed that the mechanisms responsible for behavioural and physiological responses to threat may be distinct from those underpinning the conscious emotional experience itself (a feeling). To examine this, the researchers had older adults stand on the edge of a raised platform and were stratified based on whether they reported fear in response to this postural threat. Behaviours indicative of postural “stiffening” during the threat condition were observed regardless of whether participants reported fear or not. The authors state that self-reports indicated the participants cognitively monitored these changes in balance, and fear of falling was experienced in those who interpreted these behaviours to imply that harm was likely to occur. Fearful participants also showed changes in balance, including increased movement complexity and altered utilisation of sensory feedback. The authors concluded that these behaviours were influenced by attempts to consciously control balance. As can be seen from this work, the interaction between emotional and cognitive responses (including conscious perception of fear) ultimately leads to the behavioural outcome. Those individuals who experienced the feeling of fear of falling during the study had a corresponding change to their motor control as well as alterations in sensory feedback. Autonomic responsivity (e.g., increased respiration or heart rate) that may occur in individuals who experience fear when standing at the edge of an elevated platform, can have a direct modulatory effect on the activity of the thalamic nuclei and this has implications for the way sensory information is processed and/or relayed by the thalamus to other regions of the cortex and subcortical areas (53). From this perspective, it is possible that participants in the Ellmers study who interpreted the behaviours indicative of “postural stiffening” as potentially harmful, also had more pronounced shifts in utilisation of sensory feedback, resulting in altered interpretation of the incoming sensory information (demonstrating how cognitive-emotional state impacts behavioural outcomes). This has the potential to result in high vigilance and over-sensitivity to environmental signals which are reflected in inappropriate emotional responses and ANS dynamics.

##### 4.2.2.7. Association between social evaluation threat, emotional state and postural control

Furthermore, social evaluation threats (such as the presence of an expert evaluator) have been used to elicit changes in emotional states. Doumas et al. (119) examined whether similar stress-related changes in postural sway can be observed using stress induced by social evaluative threat while performing arithmetic tasks under a time pressure. Postural sway amplitude was greater and reaction times faster when performing arithmetic task under a time pressure, but not under other task challenges. In this example, there are likely direct (i.e., arousal mediated changes, altered sensory gain, context specific influences on ones sensorimotor processing, etc.) and indirect (i.e., altered attentional allocation) mechanisms occurring which ultimately lead to the observed behavioural outcomes.

#### 4.2.3. Affect, arousal and postural control

In addition to manipulations of postural threat, studies have also explored the relationship between mood, affect and the control of posture. A common approach in neuropsychosociological research is to provoke changes in arousal and affect using picture/images. There exists, as example, an International Affective Picture System (IAPS) that is used to provide visual images for assessing emotional outcomes and these images are categorized based on the valence (negative to positive) and impact on arousal (calm to exciting) (120). The link between postural control and visual stimuli had been reviewed in detail by Lelard et al. (121). Lelard et al. (121) summarized that the majority of studies have focused on differences in valence (positive versus negative) and linked postural changes to approach and withdrawal behavior. There have been some studies who have also reported freezing in response to the most negative/arousing stimuli. As an example, Stins et al. (122) used pictures to provoke state changes in affect with images linked to positive or negative valance and measured the impact on balance control. While they noted little difference in postural sway measures across visual stimuli that were intended to provoke different emotional states, they did reveal two key outcomes. The first was a reduction in sway path when viewing pictures of mutilation that they hypothesized was fear induced bradykinesia. Second, this influence was only evident in the more challenging postural task condition (unipedal stance versus standard stance). Similarly, Roelofs et al. (123) reported significant decrease in the sway in response to the angry expressions as opposed to happy and neutral expressions and interpreted the reduced body sway as “freezing behaviour.” They suggested that freezing behaviour enables an individual to first detect relevant information, then to mobilize the whole body, and ultimately to trigger “fight or fight” behaviour (124). The challenge in such work is distinguishing the potential impact of valance versus arousal on postural control, since such strong stimuli with negative valance are also highly arousing. Horslen and Carpenter (125), sought to distinguish the effects of arousal and valance on balance control since differences associated with altered affect (positive or negative) may be attributable to changes in arousal/anxiety. Measures from both balance (COP) and ANS reactivity (EDA) were influenced by the changes in arousal independent of evoked changes in valence. Consistent with studies elevating arousal through changes in threat, the authors concluded that arousal may be the underlying mediator of emotional state related changes in postural control. While there appears to be evidence of the impact of emotional visual stimuli on postural control and that the influence of arousal state may be most important with effects of valance being inconsistent or absent. The observation that some stimuli, such as those with high negative valence evoke change in postural control may arise from indirect influences such as altered body position or even changes in muscle tone, though the latter is rarely measured. Continuing to disentangle the impact of valence versus arousal and to establish the specific mechanisms of modulation of posture control will be important to better understand the insight that can be gain from these paradigms.

### 4.3. Disorders of anxiety and emotion processing and potential links to postural control

The majority of research in the area of emotional state and postural control has been conducted in healthy individuals whose cognitive-emotional state is influenced transiently by varying task conditions. An alternative approach is to study postural control in individuals with cognitive-emotional state disorders. Unfortunately, by comparison to work with healthy adults, there is considerably less research that has explicitly examined the impact of disorders of emotional processing on balance control. In addition, a significant focus in studies on healthy individuals is directed towards states of heightened arousal where states of low arousal can be a frequent consequence of specific neurologic disorders. Furthermore, several disorders are characterised by muted autonomic or emotional reactivity which may have implications to the control of balance. Below is a brief discussion of some of the evidence of the links between the control of postural equilibrium and disorders of anxiety and emotion processing.

Balaban and Thayer (24) presented a detailed framework for neuroanatomical underpinnings of the links between anxiety and the control of balance highlighting support for a bidirectional influence. Evidence in support of these foundations has been reinforced through studies focussed on balance control among individuals with anxiety disorders (126–128) and findings have shown evidence of altered balance control among those diagnosed with anxiety disorders [e.g., generalized anxiety disorder (GAD), social anxiety disorder, or other specific phobias]. For example, Redfern et al. (129) highlighted increased sway among individuals with GAD in response to visual motion. Findings were attributed to the inability of GAD patients to disregard misleading visual information. Evidence of increased postural sway has also been observed in highly anxious children relative to children without an anxiety disorder (129). However, the relationship between anxiety and control of balance is complex as there is also evidence, in support of the experiments performed at height, that anxious individuals may adopt a defensive stiffening strategy and be characterised by lower levels of measured postural sway (130). The commonly reported increase in muscle tone that is linked to anxiety disorders such as GAD (131) is a potential indirect influence on the control of balance, as could be attentional focus. Evidence suggests shared neuroanatomical and neurochemical links as the foundation for comorbidity of balance and anxiety disorders (126).

Emotion processing deficits are often linked to challenges in regulating emotions and attentional biases that impact perception and impaired recognition of emotions, among others, and diagnosed individuals are often highly co-morbid (132). For example, persons diagnosed with alexithymia have difficulty identifying and describing feelings however it is common to be reported alongside other co-morbid conditions including neurological disease such as Parkinson’s disease (133). There exist many neurologic diseases/injuries that present with deficits in emotion processing and with balance control. Examples include Parkinson’s diseases (134), Alzheimer’s disease (135), post-traumatic stress (136) and traumatic brain injury (137). However, all are commonly characterized by other sensorimotor and/or executive processing symptoms that may also impact postural control. As a result, it is often difficult to isolate an independent association to postural control and emotion processing, and we are not aware of specific studies that have isolated a specific and independent link between emotion processing deficits and the control of balance.

While there are certainly a range of potential factors that might link these disorders to balance control there is some commonality among these disorders which may tie directly to the control of balance. There appears to be a common characteristic of altered autonomic activity among those with disorders of anxiety and emotional processing. As examples, electrodermal hypoactivity appears to be a reliable feature of depression with skin conductance measures being described as low or flat, reflecting both low tonic levels and a muted reaction to stimuli (138, 139). Anxiety disorders have been associated with muted ANS reactivity which has been described as decreased autonomic flexibility. Diamond and Fisher (140) revealed evidence of decreased ANS flexibility among those with GAD during structured interviews. It should be qualified that there are mixed findings in terms of the direction/amplitude of these effects among depressed/anxious patients which might be dependent on the type of stressor used to provoke the ANS reactivity (141). Muted ANS reactivity to arousing stimuli has also been observed among those with alexithymia (142). Among a study of those with emotional dysregulation associated with borderline personality disorder, there was a muted skin conductance and heart rate responses to emotional stimuli (143). Autonomic dysfunction is commonly reported among older adults and those with neurodegenerative disease (144). It is specifically common among those with Parkinson’s disease (145) who are also characterized by blunted ANS reactivity to emotional stimuli (127). There is of course a strong direct connection between autonomic dysfunction and balance control when considering changes in vagal tone during postural transitions associated with orthostatic hypotension (146). Such observations of attenuated resting vagal modulation and vagal reactivity in response to postural maneuvers has been observed in people with GAD (147). Given the coupled activation of ANS and somatic NS in balance control, highlighted earlier in this review, it raises the possibility that muted ANS activity, linked emotion processing disorders, may have an impact on postural control.

There is also the question of the potential association role between emotional state and vestibular disorders, the latter often considered classically as balance disorders. There is a longstanding recognition of the association between outcomes such as anxiety, fear and avoidance with dizziness and vestibular disorders (148, 149). While anxiety can be expressed among those with non-vestibular causes of balance control deficits, there is a unique relationship between vestibular function and emotional state. As reviewed by Rajagopalan et al. (150) the vestibular nuclei project widely to many centres including those involved in emotion processing and as a result, vestibular stimulation can directly influence emotional state and has even been used to treat some cognitive-emotional disorders. The direct evidence of vestibular stimulation impacting emotional state is suggested to result from shared neural networks between vestibular and emotion processing (151). The vestibular system is also a critical modulator of autonomic activity through vestibular-autonomic networks (152) which could impact not only vestibular-autonomic reflexes such as those for orthostatic regulation but also emotional responsiveness. Given the important influence of vestibular activity has across the CNS impacting cognitive, emotion and autonomic processing, there is a need to consider the consequence of vestibular dysfunction as more than simply a loss of the sensory contributions to orientation and stability control (153).

## 5. Recommendations for future studies assessing balance control

### 5.1. The need for assessment of cognitive-emotional state

As is evident through this paper, there are many research studies that demonstrate a link between emotional states, autonomic nervous system ANS activity and somatic NS activity during movement/postural control tasks. It is argued by some that the underlying association between arousal and ANS reactivity may be largely mediated by the link between emotional state and the control of balance. While we would support the idea that measuring the level of arousal and autonomic reactivity is critical to understanding balance control, this review has also highlighted links between feelings, emotional states, and postural control that may be considered independent of the direct state of arousal or ANS activity. Cognitive-emotional state, in addition to arousal, remains an important consideration within the field. The evidence of the important modulatory influence of cognitive-emotional state on balance control does raise the general concern of the need to provide such context when interpreting balance reactions and/or the outcomes of balance control studies. Many have argued for the importance of quantitative measures of balance control including kinetic and/or kinematic measures. Such fidelity is important, but the general lack of concurrent attention to the state of the individual during the test of balance, either through complementary measures and/or task controls, limit the value of such detailed measures of motor behaviour.

### 5.2. The need for novel, standardized methods to address cognitive-emotional state

Moving forward, efforts must be made to determine/standardize methods of assessing or controlling for cognitive-emotional state during balance testing. This includes addressing indirect factors such as body posture, ventilation and focus of attention, as well as situations and personal characteristics that have the potential to influence cognitive-emotional state and ultimately impact postural or balance assessment. Factors such as what the participant was doing prior to arriving for postural or balance assessment, whether the individual has had their balance/posture assessed previously, caffeine intake (and other substances that alter ANS activity), medical history that may impact emotional state, social support, etc., also need to be considered. It is also important to consider the concurrent impact of the relative task difficulty on both balance control and emotional state, and the possible interaction with somatic control. Given the information this review has presented, on both the association of emotional state, arousal and postural control and balance, and the fact that these factors are influenced by having an observer/evaluator present, it is imperative that these factors be taken into consideration during both research and clinical balance assessments.

### 5.3. The use of self-reported indices of affect and mood as insight into cognitive-emotional state

It is considered important to advance the use of tools that can provide insight into an individual’s emotional state and level of arousal during postural control tasks. There are self-reported indices of affect or mood that have been used such as the positive and negative affect schedule (PANAS), brief mood introspection scale, multiple affect adjective checklist revised version (MAACL-R), profile of mood states (POMS), and discrete emotions questionnaire (DEQ) (154). The concern is that such measures may not be sensitive enough or do not measure context during the balance task. They may also be prone to errors/biases as they are based on self-reporting. Alternatively, there is a strong case to be made for assessing the physiological responses that may be linked to cognitive-emotional state as a continuous time-varying signal that can be associated with the balance control task. There are several possible candidate signals including electrodermal activity (EDA), heart rate variability (HRV), and electroencephalography (EEG), which will be briefly discussed below.

### 5.4. The use of EDA to probe arousal and gain insight into the relationship between cognitive-emotional state and postural control

Electrodermal recordings have been the more popular research tool to use as a proxy of sympathetic activity due to their utility in representing a continuous variable that can be temporally coupled with the onset of relevant events, such as instability or perturbation (93). There are some limitations related to the temporal lag and other factors that can impact skin conductance (e.g., body temperature) but the methodology and analytics are standardized and could be more broadly introduced. However, use of EDA alone, as a proxy for arousal, does not meet the need for considering an individual’s cognitive-emotional state prior to and at the time of testing.

### 5.5. The use of HRV to provide insight into the relationship between cognitive-emotional state and postural control

HRV is an index of sympathetic and parasympathetic tone which has been used to a limited extent to examine the relationship between emotional state, autonomic and somatic activity, and postural control (155, 156). Among other outcomes, HRV has been found to correlate with cognition, attention, reaction time, memory, emotional regulation, postural control and executive function (155–157). It has also been found that different emotional states are reflected in individuals’ state-specific patterns in heart rhythms (158), independent of the amount of HRV, although state-specific changes in the magnitude of HRV are also important. Having high heart rate variability (HRV) is associated with higher emotional well-being, including being correlated with lower levels of worry and rumination, lower anxiety, and generally more regulated emotional responding (159–164). Thus, individuals with higher HRV appear to be better at regulating their emotions. Furthermore, cardiac coherence has been found to be related to emotional states (158). Using a 24 h index of HRV to help inform the emotional regulation of each individual and a 5 min HRV measurement prior to and following research collection, as well as during collection, to quantify the interaction between situation-specific changes and potential balance perturbation (i.e., stimuli coming from the environment). These measures, along with others introduced here, would enable future studies to examine whether individuals who are better able to regulate their emotional state are also less affected by an emotionally triggering stimulus in the environment (such as postural threat) and to assess the impact the stimulus on balance and postural outcomes. Given correlations of HRV and emotional state, and that it is an index of ANS activity, HRV could be a useful tool to utilize when examining the relationship between emotional states, ANS activity and postural control.

### 5.6. The use of EEG to provide insight into the relationship between cognitive-emotional state and postural control

In addition to HRV, EEG recordings would be beneficial to examining this relationship [please see reference (165) for a review of EEG computation methods for emotional state]. It has been found that changes in the demand to balance control are associated with changes in activity of the cortex as measured by frequency content of EEG recordings (166, 167). Many studies have shown cortical contributions to the recovery of balance following a perturbation in humans (168–171). Event-related potentials (ERP) are known to reflect underlying emotional responses when emotional stimuli are present (i.e., a threat or other emotional stimuli) (165). Furthermore, analysis of cortical cardiac coherence (as well as cortical coherence) could prove to be important when examining this relationship, when considering that 85%–90% of the fibers making up the vagus nerve are afferent, and cardiovascular related afferent neural signal transmission significantly affects activity in most higher brain centers, as well as cognitive processes and emotional state (158, 172, 173).

### 5.7. Future studies

Finally, future studies should explore these relationships among individuals with ANS, mood and emotional processing disorders for their potential links to postural control, as well as in individuals with neurologic disorders (e.g., Parkinson’s disease) where pathology can impact cognitive-emotional processing and autonomic centres. Experimental models that look at the impact of disordered emotion and ANS control on balance reactions should extend beyond the effects of fear/anxiety and threat to include a wider breadth of emotional states and their influence on postural control. Doing so will help to gain understanding of the complex interplay between central and peripheral components that create the conscious perception of our emotional state and how this relates to postural control.

## 6. Conclusion

- There are strong links between cognitive-emotional state, ANS activity and somatic NS activity in postural control supported by shared neuroanatomical networks including integrated involvement of the limbic system.
- Support for the interaction between cognitive-emotional state and balance control emerges primarily from behavioural studies exploring the impact of evoked changes in emotional state, most often using threatening states, on measures of postural control such as postural sway.
- However, there remains a need to increase a focus on these important associations among those with disordered/altered emotional state, ANS processing and vestibular function.
- It is important to distinguish whether postural behaviours resulting from changes in cognitive-emotional state are from direct or indirect sources to better advance understanding of the specific associations between emotional state, ANS and somatic NS activity and postural control.
- There is a need for development of standardized methods to assess cognitive-emotional state in postural control studies to advance fundamental understanding and to inform interpretation of posture control behaviour. The most promising are likely based on biophysical signals such as EDA, ECG and/or EEG.
- It remains important to further our understanding of the influence of cognitive-emotional states on postural control in a clinical setting for both assessment and treatment of postural and balance dysfunction.

## Author contributions

KH wrote the first draft of the review paper. WM and KO wrote sections of the manuscript. All authors contributed to the article and approved the submitted version.

## Funding

This is work is support by funding from the Natural Science and Engineering Research Council of Canada (NSERC).

## Conflict of interest

The authors declare that the research was conducted in the absence of any commercial or financial relationships that could be construed as a potential conflict of interest.

## Publisher’s note

All claims expressed in this article are solely those of the authors and do not necessarily represent those of their affiliated organizations, or those of the publisher, the editors and the reviewers. Any product that may be evaluated in this article, or claim that may be made by its manufacturer, is not guaranteed or endorsed by the publisher.
